# Nursing Workforce Retention in Rural Ghana: The Predictive Role of Satisfaction, Rural Fit, and Resilience

**DOI:** 10.1155/2023/9396817

**Published:** 2023-02-21

**Authors:** Collins Atta Poku, Eva Mensah, Josephine Kyei, Adelaide Maria Ansah Ofei

**Affiliations:** ^1^School of Nursing and Midwifery, University of Ghana, Legon, Accra, Ghana; ^2^Department of Nursing, Kwame Nkrumah University of Science and Technology, Kumasi, Ghana

## Abstract

**Introduction:**

High turnover of nurses in rural healthcare settings contributes to challenges in healthcare delivery. Various incentive packages have been introduced in rural healthcare settings to curb this phenomenon, but the canker still exists. The study aimed at assessing the predictive role of job satisfaction, rural fit, and resilience on nurses' retention in rural Ghana.

**Materials and Methods:**

A multicentre cross-sectional design was adopted to collect data from 462 nurses. Analysis through descriptive statistics, one-way ANOVA, Pearson moment product correlation, and multiple regression was done.

**Results:**

There was low resilience and rural fit among nurses with higher turnover intention, which was predicted by average daily attendance (*β* = 0.108), rural fit (*β* = −0.144), resilience (*β* = −0.350), satisfaction with prospects (*β* = −0.187), and satisfaction with prospect and pay (*β* = −0.171) at the significance of 0.05.

**Conclusion:**

Policymakers can be assured that not just improving financial incentives to nurses, but the integration of nurses to rural settings, commensurate workload and improving pay and prospects for professional growth and resilience are needed for rural retention. Implications for nursing management, nurse managers, and policymakers have a role to develop sustainable strategies to integrate rural fit, resilience, and job satisfaction to help reduce turnover among nurses.

## 1. Introduction

Human resources for health care are among the six pillars of the health system. Health systems can significantly be effective when there is a satisfactory health workforce, thus, the key to improving health service coverage and outcomes, although multifaceted, depends on the adequacy and skill mix of the workforce [1].

Globally, there is an increasing worry about human resource shortages in rural healthcare settings, especially, in low- and middle-income countries (LMICs) with Sub-Saharan Africa (SSA) experiencing the severest form. The capacity of the health workforce in these regions is insufficient to meet the population's health objectives [2]. The health system is confronted with health workforce shortages, and this disparity is an important limitation in realizing the health-related sustainable development goals (SDGs) [3]. Meanwhile, the need for more workforce is projected to significantly go up due to multiple factors: adolescent-related challenges, ageing of the current generation, changing distribution of diseases, high incidence of noncommunicable diseases (NCDs) resulting from a sedentary lifestyle, overpopulation, and high pace of the technology-induced world [4]. The strain on the workforce is imminent if the challenges on the workforce are not addressed, and therefore, plans are needed for the management of human resources in the health sector [5].

In the interim, there has been an estimated shortage of nine million nurses, and this figure is projected to increase further by two million by 2030 with a disproportionate effect on the SSA [6]. Moreover, the problem of the low number of nurses in rural areas is further aggravated by absenteeism, with its glitches of shortages, skill mix disparities, and unfair distribution [7], increased turnover [8], inadequate specialised knowledge-base of some providers [9], and poor work environment [10].

The uneven human resource distribution in rural areas is the foremost problem that is most noticeable in the nursing workforce which is considered the linchpin of the health system responsible for providing the bulk of healthcare services to its clients [11].

The major contributor to this shortage is workforce turnover; which is expressed as the percentage of the workforce that has stopped their job. Park and Yu [12] highlight the multidimensional factors that impact the turnover of nurses in rural health facilities. Turnover is noted to have a significant impact on the health system, as an average of $37,000 to $67,000 is paid for replacements [13] while retention minimizes financial costs and boosts good patient outcomes [14].

Nurses' turnover intentions are influenced by so many factors [15]. The COVID-19 pandemic has worsened the situation as a result of increased migration due to the global shortfall. There is a range of reported cases of job dissatisfaction, challenges to patient and workforce safety, burnout, and unfavourable scheduling in rural settings [16] with regions with low densities of workforce producing the worst health outcomes [17].

The challenge of retaining nurses in rural areas of Ghana still poses a challenge [18]. To improve the status of overworked nurses and patient outcomes, effective execution of human resource management methods is required [19]. Though many strategies [20] have been instituted in the past to address the problem of workforce disfavouring in rural areas in Ghana, there are still challenges to the rural retention of nurses. The study, therefore, assessed the predictive role of job satisfaction, rural fit, and resilience on nursing workforce retention in rural areas.

## 2. Materials and Methods

### 2.1. Design

A cross-sectional descriptive survey was employed to determine factors that predict nursing workforce retention in rural healthcare settings in Ghana.

### 2.2. Study Setting

The study was conducted in selected facilities in the Ashanti region, a region which lies in the middle belt of Ghana. According to the 2014 Demographic Health Survey conducted by the Ghana Health Service (GHS), almost half of the inhabitants reside in rural areas [21]. The region has 530 facilities, comprising government (170), mission (71), private (281), and quasi-government (8) facilities, with 48.7% of the facilities found in rural districts. It has a nursing workforce of 11,412, with about half in rural districts [22]. A district is considered rural and deprived if it lacks basic amenities for its populace, has low-performing health indicators and human resource retention, and is also difficult to reach through transportation and Internet connectivity. Out of the 43 districts in the region, fourteen (14) have a rural population exceeding the urban population and, therefore, classified as rural [21].

### 2.3. Study Population

The population included registered nurses (RNs)—general nurses and specialist nurses in rural healthcare facilities. All RNs with permanent postings to their facility were included in the study while RNs who had not worked for at least 6 months at the present post were excluded. The data collection was carried out between May and August 2022.

### 2.4. Sampling and Sample Size

Based on Slovin's formula, a total of 571 RNs was estimated, using a margin of error of 4% and a confidence level of 95% and recruited for the study [23]. The participants were sampled through a multistage sampling approach. Five districts out of the 14 rural districts were selected randomly once they satisfied the criteria for the classification in the region. A simple random sampling was done to select five health facilities from each of the five selected districts (25 facilities overall). A proportionate stratified sampling approach based on the numerical strength of the nursing workforce of the 25 facilities was done. Convenience sampling was used in recruiting the participants throughout the 25 facilities by the researchers.

### 2.5. Measures

Sense of Community Index II, the Brief Resilience Scale, three subscales of the Measure of Job Satisfaction Scale, and the Turnover Intention scales were used to measure the variables under study.

#### 2.5.1. Rural Fit

A 24-item Sense of Community Index II (SCI II) was used to measure the rural fit of RNs and; thus, how RNs felt about their location of work [24]. Responses for items ranged from 1 = *strongly disagree* to 5 = strongly agree on a 5-pointLikert-type scale. A composite mean score of 2.5 and above indicated higher levels of rural fit. Studies have used the tool and have reported Cronbach's alpha between 0.75 and 0.85 [25]; in this study, it was 0.79.

#### 2.5.2. Resilience

The Brief Resilience Scale (BRS), which is a 6-itemself-report measure, was used to assess RNs' resilience [26]. The scale measures the ability to bounce back or recover from stress. Participants rated each item on a Likert scale of 1-Strongly disagree to 5-Strongly agree. The score is computed through the average of the six (6) items. Composite mean scores above 2.5 indicate RNs' high resilience among RNs. BRS has been used in many studies and has reported Cronbach alpha scores of 0.80 to 0.95 [27], the BRS presented a high internal consistency (Cronbach's *α* = 0.88) in this study.

#### 2.5.3. Satisfaction

The study used three subscales (satisfaction with workload (8 items), satisfaction with professional support (8 items), and satisfaction with pay and prospects (10 items)) from the Measure of Job Satisfaction scale [28]. Items were scored on a 5‐point Likert scale (1 = very dissatisfied and 5 = very satisfied). A score for the scale was done by summation of individual items. Composite scores above 2.5 were considered as higher job satisfaction. The scale has been used in many studies and has reported higher reliability coefficients between 0.80 and 0.93 [29]. The internal consistency in the present study was 0.89 (overall scale), 0.91 (satisfaction with the workload), 0.78 (satisfaction with professional support), and 0.84 (satisfaction with pay and prospects).

#### 2.5.4. Turnover Intention

A six-item Turnover Intention scale designed from scales by Mobley et al. [30] and Ganesan and Weitz [31] was used to measure RNs' turnover intention. The items were measured on a five-point scale; from 1 = “Strongly disagree” *to* 5 = “Strongly agree.” A score above 15 indicated higher turnover intentions. The internal consistency coefficients for the scale in other studies ranged between 0.80 and 0.90 [32]. The Cronbach's alpha for the scale after the pre-test was 0.84.

### 2.6. Data Analysis

The data was analysed using SPSS version 26 through descriptive and inferential statistics at a significance of 0.05. Descriptive statistics were conducted on participants' demographics. Associations between turnover intention, average patient attendance, rural fit, resilience, and RN satisfaction were analysed using Pearson's moment product correlation, while differences in turnover intention scores between certificate, diploma, and bachelor degree nurses were tested using one-way ANOVA. A multiple linear regression analysis using the standard approach was conducted to predict nurses' turnover intention in the rural setting.

### 2.7. Ethical Consideration

Ethical approval was sought from the Institutional Review Board of Noguchi Memorial Institute of Medical Research (IRB-NMIMR CPN 012/21-22). Permission was obtained from the management of the selected facilities. Additionally, participants' anonymity and confidentiality were assured.

## 3. Results

### 3.1. Sociodemographic Characteristics of Participants

A total of 462 participants responded to the scale (response rate of 80.9%). The results on socio-demographic characteristics of participants in the study showed that the majority of the participants (66.5%) were below 30 years, unmarried participants (60.2%), had a diploma (58.4%), worked at the wards (44.8%), and were paid below US$500 as a monthly salary (69.3%). Details on sociodemographic data are provided in Table 1.

### 3.2. Job Satisfaction, Rural Fit, Resilience, and Turnover Intentions among Nursing Workforce

The findings on the nursing workforce's satisfaction, rural fit, resilience, and turnover intention in rural healthcare settings are presented in Table 2. The composite mean score of RN's satisfaction was 2.81, satisfaction with professional support (*n* = 3.13), satisfaction with pay and prospects (*n* = 2.01), and satisfaction with workload (*n* = 3.28). Moreover, the composite mean scores of resilience (*n* = 2.33) and rural fit (*n* = 2.10) meant “low resilience” and “low rural fit,” respectively. On the extent of nursing workforce turnover intentions in rural healthcare settings, a composite mean of 2.83 indicated a high turnover intention.

### 3.3. Comparison of Turnover Intention among Categories of the Nursing Workforce

A between-subjectone-way ANOVA was performed to compare the turnover intentions among nursing workforces' highest qualifications (certificate, diploma, and bachelor degrees). The results as shown in Table 3 which showed a statistically significant difference in turnover intentions (*F*_(2,459)_ = 12.635, *p* < 0.001) among the various categories of the nursing workforce in rural healthcare settings. A follow-up post hoc analysis using Tukey's HSD (*α* = 0.05) showed that the nursing workforce with bachelor's degrees had higher turnover intention compared to those workforces with a certificate [MD = 3.783, *p* < 0.000] and diploma (MD = 3.704, *p* < 0.000). Although nurses with diplomas had high turnover intentions than certificate holders (MD = 0.075, *p* > 0.05), the difference was not statistically significant.

### 3.4. Relationship between Work Characteristics, Rural Fit, Resilience, Satisfaction, and Workforce Retention in the Rural Health Setting

The Pearson product-moment correlation determining the relationship between turnover intention, average daily attendance, rural fit, resilience, and satisfaction of the RNs in the rural healthcare setting is shown in Table 4. There was a significant positive correlation between turnover intention and average daily attendance (*r* = 0.131). There was, however, a statistically significant but negative correlation between turnover intentions and resilience (*r* = −0.357), rural fit (*r* = −0.168), and satisfaction with pay and prospects (*r* = −0.187). This implies that nurses turn to quitting their job or profession when resilience is low. Similarly, inadequate rural fit and dissatisfaction with pay and prospect are linked with increased turnover intentions.

### 3.5. Predictive Effects of Satisfaction, Resilience, and Rural Fit on RNs' Turnover Intention in the Rural Healthcare Settings

Multiple linear regression was used to evaluate the effect of average daily attendance, satisfaction, resilience, and rural fit on nurses' turnover intention in rural settings. The result predicting turnover intention is shown in Table 5. The model was significant, predicting 21.9% of the turnover intention among RNs (*R*^2^ = 0.219, *F*_(5,456)_ = 25.59, *p* < 0.001). When all variables were considered in a single model; average daily attendance by RNs (*β* = 0.108), rural fit (*β* = −0.144), resilience (*β* = −0.350), and satisfaction with pay and prospects (*β* = −0.187) remained significant predictors.

## 4. Discussion

The purpose of the study was to investigate the relationship between rural fit, resilience, and satisfaction of the nursing workforce on nurses' turnover intentions in rural healthcare facilities. The findings indicate that several strategies have significant correlations with subsequent rural retention. Nurses' satisfaction, rural fit, resilience, and average daily attendance of patients contribute to future rural retention, and policymakers can be confident of overcoming the challenge if these determinants are addressed.

The study findings indicated that nurses with bachelor's degrees are more likely to leave their jobs than their counterparts with diplomas and certificates, and this position is supported by Fontes et al. [33]. This may be due to inefficient communication about conditions that enhance personal fulfilment, professional success, and the delivery of high-quality nursing care. Moreover, the high workload in rural settings combined with low pay and lack of prospects for professional growth could be blamed for the phenomenon [34, 35]. Additionally, the fact that most rural hospitals have local conditions where nurses are exposed to poor working conditions and few career chances influences turnover intention [36].

Consistent with Holland et al. [37], the findings of this study posit that a perceived workload had a detrimental effect on nurses' satisfaction, with a resultant increase in turnover intention. The results show that workplace policies that prioritize the workload of nurses can reduce turnover.

In other domains, nurses believe that the combination of financially viable incentives, such as hardship allowances and free transportation are adequate to cover the opportunity costs related to working in remote locations [38]. The finding of this study indicated that satisfaction with pay and prospect by most nurses serves as a motivation to remain at the post. While the staff are encouraged to stay in rural places, it is important to prioritize higher compensation and benefits [12]. The major challenge, however, is the disregard for the WHO recommendations on rural incentives for health worker motivation in rural areas [20], which are not being implemented. Although in individual countries, there are policies for rural incentives in the form of allowances; they are either not paid or insufficiently paid [39]. Salary and allowances are two of the major elements that affect health professionals' decisions to remain in rural employment. However, overreliance on financial incentives to curtail high turnover among rural nurses may be a smokescreen as other important strategies should equally be considered [40]. Yong et al. [41] found that nurses in rural settings are not influenced to remain by financial incentives, and suggested that any monetary incentives should be targeted only to those whose decisions to practice in rural settings are persuaded by money. Increasing wages and good working conditions are significant inducements for nurses to stay in the health industry in many LMICs, raising wages in environments with limited resources has, however, been a challenge; it is therefore, critical to employ a variety of solutions to solve the shortage of nurses in LMICs [42].

Moreover, the findings of the study highlighted that nurses who find themselves rurally fit are willing to remain in the post. Graduates' likelihood of returning to practice in rural areas is increased by their rural upbringing as personnel from rural backgrounds continue to work in rural areas for a decade or more years on average after passing out [43, 44]. In a similar vein, recruiting staff for their home region has yielded a positive response, as support from family and friends has benefited them and increased retention [45]. When considering models of rural nursing retention, this finding offers additional help for understanding how the nurse is integrated into the community environment. The results highlight the significance of rurality's cultural aspects as nurses are not content with their work unless content with the rural setting. Unfortunately, people who like the leisure activities and lifestyles of urban locations are likely to have increased turnover intentions [46].

As a means of improving rural retention, policymakers should consider the development and implementation of the professional programme as recommended by the WHO on rural retention. Thus, nurse managers can assist nurses to advance their careers through study leave policies and flexible training schedules. Such arrangements have been successful as reported in other settings, which discovered that employment in the rural public sector was more alluring given the possibility of receiving health sponsorship or scholarships [47]. However, to encourage nurses' trust in the system of career advancement, strong adherence to the policy's implementation is required, such as establishing open procedures to end corruption in administrative promotion would inspire providers to follow this course for their professional advancement.

Constraints of rural practice, such as poor infrastructure, lack of recreational activities, limited lodging facilities, work overload, and feelings of isolation, may serve as unfavourable indicators of retention [48]. The capacity to put up with these constraints is the panacea for the retention of nurses. People with high levels of resilience can endure these constraints and make meaning out of the situation [49]. The results of this study accordingly support the idea that traits of resilience are likely to help nurses' ability to survive and remain in rural settings for long periods. These findings are significant for workforce initiatives in rural areas because they give us a foundation for understanding resilience. The idea has been suggested in the past that building resilience assists nurses to overcome burnout and lower turnover expenses [50]. On contrary, it is also likely that nurses who find it difficult to recover from work stresses may consider quitting to solve their issues. If this is the case, encouraging nurse resilience might be one tactic to reduce turnover. Additionally, a poor nurse-job fit could weaken the resilience of nurses [47]. Posting of nurses to rural areas should be done cautiously taking into account all the factors enumerated in this study to enable the retention of RNs.

## 5. Limitation

The cross-sectional nature of the study makes it difficult to infer causal relationships. Again, the data was all gathered by self-report, and this could have exaggerated the connections between the variables. Only part of the Measure of Job Satisfaction scale was used; this made a comparison with previous studies that used the whole scale problematic. The researchers, however, discussed the job satisfaction of RNs based on the individual subscales. Future research should consider a qualitative study to explore the nature of resilience and job satisfaction among nurses, and this can help the course of rural retention.

## 6. Conclusion

Though there is a growing corpus of research available on strategies to increase nurses' retention in rural settings, policymakers can be assured that not just improving financial incentives to nurses who work in rural settings, but taking into account the background of nurses, improving the prospects for professional growth, nurses' resilience, and removing obstacles connected with a longer stay in rural areas can improve nurses' retention.

### 6.1. Implication for Nursing Management

The retention of the rural workforce depends on activities to integrate rurality into health staff living conditions, increase nurse-job satisfaction, and build resilience. These findings give decision-makers more assurance and broaden their arsenal of potential actions to increase retention, offset significant financial and human costs and promote nurses' interests to better fit their communities. We contend that effective management of the nursing workforce, specifically through a satisfactory practice environment and the provision of organizational assistance via professional support, should be a key component in the development of a skilled nursing workforce in the healthcare industry.

Nurse managers should also come up with practical solutions to aid their nurses in blending in well with the rural environment as turnover intentions will ultimately depend on nurses' capacity to meet the demands of their work environment. Nurses succeed greatly when managers provide practical assistance, including flexible work hours, professional support, work-life balance, and support for overcoming obstacles in life. Additionally, initiatives to increase nurses' resilience, such as training programmes and cultivating a pleasant workplace culture are other successful ways to further reduce turnover intentions in rural settings. The development of healthcare policies that improve the job satisfaction of nurses (pay and prospects) and support the development of resilience and working conditions in rural settings are recommended to enhance rural integration among nurses.

## Figures and Tables

**Table 1:** Registered nurses' sociodemographic and professional information.

Variables	Categories	Frequencies (*N* = 462)	Percent
Age group	30 and below	307	66.5
	31–40	139	30.1
	41 and above	16	3.4

Gender	Male	108	23.4
	Female	354	76.6

Marital status	Single	267	57.8
	Married	184	39.8
	Others	11	2.4

Highest qualification	Certificate	132	28.6
	Diploma	270	58.4
	Bachelor degree	60	13.0

Unit of work	RCH unit	38	8.2
	Maternity unit	53	11.4
	Emergency unit/OPD	164	35.5
	Ward	207	44.8

Level of salary	Below $500	320	69.3
	$500–$750	139	30.1
	$750	3	0.6

**Table 2:** Nursing workforces' satisfaction, rural fit, resilience, and turnover intentions.

Variables	Mean	SD
Satisfaction	2.81	0.88
Satisfaction with professional support (8 items)	3.13	0.88
Satisfaction with pay and prospects (10 items)	2.01	0.87
Satisfaction with workload (8 items)	3.28	0.88
Rural fit (24 items)	2.10	0.58
Resilience (6 items)	2.33	0.63
Turnover intentions (6 items)	2.83	0.92

**Table 3:** Comparison of nursing workforce turnover intention in terms of the highest qualification.

		Sum of squares	df	Mean square	*F*	Sig
Turnover intention	Between groups	727.784	2	363.892	12.635	0.000
	Within groups	13219.169	459	28.800		

*Post hoc analysis*						
Variable	(*I*) highest qualification	(*J*) highest qualification	Mean difference (*I*-*J*)		Std. error	Sig
Turnover intention	Certificate	Diploma	−0.07593		0.56995	1.000
		Bachelor degree	−3.78333^*∗*^		0.83557	0.000
	Diploma	Certificate	0.07593		0.56995	1.000
		Bachelor degree	−3.70741^*∗*^		0.76594	0.000
	Bachelor degree	Certificate	3.78333^*∗*^		0.83557	0.000
		Diploma	3.70741^*∗*^		0.76594	0.000

*p* value <0.05.

**Table 4:** Correlation between turnover intention, average daily attendance, rural fit, resilience, and satisfaction of the nursing workforce.

Variables	1	2	3	4	5	6
(1) Turnover intention	1					
(2) Average daily attendance	0.131^*∗∗*^	1				
(3) Rural fit	0.168^^*∗∗*^^	0.014	1			
(4) Resilience	−0.357^*∗∗*^	−0.035	−0.289^*∗∗*^	1		
(5) Satisfaction with pay and prospects	−0.187^*∗∗*^	−0.014	0.101^*∗*^	−0.042	1	
(6) Satisfaction with workload	−0.127	−0.036^*∗*^	0.328	0.163^*∗∗*^	0.238	1
(7) Satisfaction with professional support	−0.230	0.038	0.201	0.288^*∗∗*^	0.312^*∗*^	0.025

^*∗∗*^Correlation is significant at the 0.01 level (2-tailed). ^*∗*^Correlation is significant at the 0.05 level (2-tailed).

**Table 5:** Multiple linear regression model testing the relationship between rural fit, resilience, satisfaction with pay and prospect, and turnover intentions of the nursing workforce.

	*B*	SE	Β	*T*	Sig
(Constant)	27.688	1.570		17.635	0.000
Average daily attendance	0.034	0.013	0.108	2.602	0.010
Rural fit	0.057	0.018	−0.144	3.190	0.002
Resilience	−0.510	0.063	−0.350	−8.059	0.000
Satisfaction with pay and prospects	−0.196	0.047	−0.187	−4.146	0.000

Model summary: *R*^2^ = 0.219, *F*_(4,457)_ = 25.59, *p* < 0.001. a. Dependent variable: turnover intentions.

## Data Availability

The data are made available from the first author upon reasonable request.
